# Supplementary material for: Target product profiles of laboratory and data analytical frameworks for genotyping to monitor antimalarial efficacy
Source: PLOS Glob Public Health. 2026 May 29;6(5):e0006500. doi: 10.1371/journal.pgph.0006500 (PMC13221082; doi:10.1371/journal.pgph.0006500)
Supplement: S1 Table — All issues listed here have a greater effect on efficacy outcomes as the proportion of study participants with recurrent infection increases (i.e., when transmission and/or recurrence rates are high). (DOCX) [file pgph.0006500.s001.docx]

| **S1 Table. *Issues negatively affecting accuracy of TES genotyping classification using currently recommended methods.*** All issues listed here have a greater effect on efficacy outcomes as the proportion of study participants with recurrent infection increases (i.e. when transmission and/or recurrence rates are high). | | | |
| --- | --- | --- | --- |
| **Issue adversely affecting estimates of treatment failure** | **Effect on treatment failure estimate** | **Factors increasing magnitude of effect** | **Proposed solutions** |
| Alleles from reinfection on Day of recurrence match those on Day 0 by chance | Overestimation | - Fewer loci  - Lower diversity of loci  - More related parasite population  - Higher COI | - Increase number and diversity of loci  - Analysis methods accounting for allele frequency, relatedness of parasites, and COI of infections |
| False positive alleles | Overestimation | - Poor specificity, e.g. due to PCR artifacts generating when amplifying repetitive regions  - Higher probability of false positive alleles matching true alleles | - Increase specificity of lab methods  - Use lab methods where false positive alleles are less likely to be the same as true alleles  - Analysis methods accounting for false positive alleles |
| False negative alleles | Underestimation | - Higher COI (resulting in more low abundance alleles)  - Larger range of allele size (PCR amplification bias for shorter alleles)  - Limited dynamic range for detection  - Misidentification of alleles due to wrong sizing of length polymorphisms | - Amplification of targets where alleles have similar length  - Increase sensitivity for detecting low abundance (minority) alleles  - Analysis methods accounting for false negative alleles  - Improve sizing or switch to NGS methods |
| Subjective genotyping methods | Inconsistent / irreproducible results | - High rates of unpredictable artifact generated by PCR of repetitive regions  - Difficulty in determining true alleles from artifact when using electrophoresis, requiring “expert” interpretation | - Lab methods with fewer and more tractable false positive alleles  - Automated analysis pipelines for data analysis |
| Inconsistent approach to analysis | Inconsistent / irreproducible results | - Simpler, match counting algorithms result in biased, inconsistent results  - Some methods difficult to implement | - Easy to use, accurate, and validated analysis tool to calculate treatment failures  - Proper accounting for uncertainty  - Automated collation of reporting data |
