# Supplementary material for: Target product profiles of laboratory and data analytical frameworks for genotyping to monitor antimalarial efficacy
Source: PLOS Glob Public Health. 2026 May 29;6(5):e0006500. doi: 10.1371/journal.pgph.0006500 (PMC13221082; doi:10.1371/journal.pgph.0006500)
Supplement: S2 Table — Minimal heterozygosity* per locus needed to achieve the classification performance of 95% specificity and 95% sensitivity determined by simulations and classification using a statistical method that accounts for complexity of infection (COI), allele frequency, and imperfect detection of minority alleles. < insert ref> For simplicity, the current simulation assumes both samples have the same COI. NA are present when a heterozygosity of 0.95 in all loci is not high enough to achieve 95% sensitivity and specificity. *Heterozygosity is a measure of allelic diversity at the locus. It is defined as the probability that two alleles taken at random from the local population are different, i.e., higher heterozygosity indicates higher diversity. (DOCX) [file pgph.0006500.s002.docx]

**S2 Table. *Guidelines for number and diversity of loci required for accurate genotype correction.***  Minimal heterozygosity* per locus needed to achieve the classification performance of 95% specificity and 95% sensitivity determined by simulations and classification using a statistical method that accounts for complexity of infection (COI), allele frequency, and imperfect detection of minority alleles.<insert ref> For simplicity, the current simulation assumes both samples have the same COI. NA are present when a heterozygosity of 0.95 in all loci is not high enough to achieve 95% sensitivity and specificity. *Heterozygosity is a measure of allelic diversity at the locus. It is defined as the probability that two alleles taken at random from the local population are different, *i.e.*, higher heterozygosity indicates higher diversity.

|  | **COI = 1** | **COI = 2** | **COI = 3** | **COI = 5** | **COI = 7** |
| --- | --- | --- | --- | --- | --- |
| **3 loci** | **0.752** | **NA** | **NA** | **NA** | **NA** |
| **6 loci** | **0.487** | **0.866** | **0.950** | **NA** | **NA** |
| **9 loci** | **0.353** | **0.739** | **0.910** | **NA** | **NA** |
| **12 loci** | **0.266** | **0.593** | **0.885** | **NA** | **NA** |
| **15 loci** | **0.221** | **0.485** | **0.839** | **NA** | **NA** |
| **18 loci** | **0.190** | **0.416** | **0.754** | **NA** | **NA** |
| **21 loci** | **0.164** | **0.362** | **0.657** | **0.950** | **NA** |
| **24 loci** | **0.145** | **0.316** | **0.582** | **0.928** | **NA** |
