# Supplementary material for: Target product profiles of laboratory and data analytical frameworks for genotyping to monitor antimalarial efficacy
Source: PLOS Glob Public Health. 2026 May 29;6(5):e0006500. doi: 10.1371/journal.pgph.0006500 (PMC13221082; doi:10.1371/journal.pgph.0006500)
Supplement: Attachment — Submitted filename: TES TPP response to reviewers bg.docx [file pgph.0006500.s005.docx]

We thank the reviewers and editorial team for their careful consideration of this manuscript. We have addressed all comments from the reviewers point by point below, resulting in what we believe to be a stronger and more accessible manuscript. We appreciate your time in considering this resubmission.

*Reviewer #1: The manuscript focuses on guidelines for genotyping correction for therapeutic efficacy studies (TES). Genotyping for TES has been challenging due to the lack of accurate methods. Genotyping in TES is focused on the question of whether infections are new versus recrudescent, and therefore whether drug treatment has been effective. In recent years methods for genotyping have evolved and become more sensitive and accurate due to availability of polymorphic marker panels and next generation sequencing methdologies. In addition analytical methods have been developed that provide information on relatedness, which is critical to differentiating between new and recrudescent infections.

 The manuscript describes the results of an expert review of methodologies and provides guidelines for applying these genotyping strategies to TES in the form of TPPs, Target Product Profiles. The evaluation included simulation of desired levels of diversity and the nos of markers required for different infection complexities, which are complicating aspects of malaria which can vary greatly at different transmission intensities. Both desired and essential characteristics are described.

 This is a timely and important publication to ensure best practice/high quality research is used for decision making. Generally the manuscript is well-written and makes a strong rationale for the TPPs for TES genotyping tools that are agnostic to the numerous genotyping panels, developed for Plasmodium. The simulation is appropriate and the TPPs are extensive and cover a large range of required characteristics. They were developed/refined by a large list of experts that this reviewer confirms are indeed global experts in malaria epidemiology and genotyping, some of whom have developed their own panels specifically for the use case described. Others are bioinformaticians/modellers familiar with the complexities and challenges of Plasmodium molecular data. While the study focuses on genotyping correction for TES, it makes an important note that these guidelines may be just as important for malaria studies and surveillance activities, including research. These guidelines could also be implemented outside of clinical trials and implemented in all research contexts.*

We thank the reviewer for their careful reading of the manuscript and reinforcement of the relevance of this work.

Specific comments

 *1) The paper provides goals for genotyping tools to sensitively and specifically classify infections , but does not show examples of how the data may be used and is therefore not accessible to a broad audience. A figure/schematic would be useful to illustrate how the TPPs will work in practice.*

We thank the reviewer for flagging the lack of a grounding point for a broad audience. We have taken the reviewer’s suggestion and now added a summative table (Table 1) that we hope provides additional context and makes the paper more accessible for a broader audience.

**Table 1. *Summary of current methods and proposed improvements to laboratory assays and data analysis methods for molecular correction.***

|  | Laboratory assay | Data analysis |
| --- | --- | --- |
| Objective | Generate genotype data from parasites from paired baseline and recurrent parasitemia samples with enough sensitivity to detect all strains that persist from baseline to day of recurrence and enough resolution to allow differentiation of unrelated strains | Analyze paired genotype data to accurately classify cases of recurrent parasitemia as either due to recrudescence or new infection |
| Scope | Wet lab assays that generate raw genetic data and bioinformatics pipelines that identify the alleles present from these data. | Algorithms that use data on alleles present to infer recrudescence or new infection state for cases of recurrent parasitemia; optional extension into statistical algorithms that also generate study arm-level failure rates |
| Current guidance | Capillary electrophoresis of three length polymorphic markers | Match-counting algorithm where recrudescence is defined by a shared allele at all loci |
| Key limitations of current methods | Poor sensitivity in detecting minor alleles; limited diversity in some settings; low accuracy; limited reproducibility | No consideration of missingness or noise in genotyping data; no incorporation of number or frequency of alleles detected *i.e.,* attempt to account for random matching; failure to estimate uncertainty in classification |
| Proposed way forward | Assess suitability of newly developed or existing genotyping methods using criteria laid out in Tables 2-4;  Validate genotyping methods using experimental approaches and/or comparison against other methods | Design new or adapt existing statistical frameworks and verify they comply with criteria laid out in Tables 5-6; Validate and compare statistical algorithms against each other using *in silico* experiments |

*2) The technical details in the main table will only be of use to those using/developing genotyping approaches – can a lay version of this table be produced to make the information more accessible to a broad audience including control programs – this will help to illuminate some of the key issues.*

We appreciate the point made by the reviewer and have added a new table (Table 1) that now serves as a “lay” summary of the content from the remaining tables.

*3) The authors make recommendation to use mixtures of P. falciparum clones at known proportions to identified sensitivity of assays but also validation of analysis methods – but do not give specific details on this. Suggest to include an example protocol/workflow.*

We fully agree with the reviewer that, after community discussion and agreement upon the TPP outlined here, the next step would be to create specific protocols aimed at evaluating methods for their adherence to the TPP. This could critically include potential QA schemes, as highlighted by the reviewer and emphasized in the manuscript. While an important step, we feel these protocols are best created by relevant bodies (such as the WHO) after discussion and possible revision to the TPP outlined here.

*4) No mention of Plasmodium vivax and the issues particular to clinical trials e.g. relapse vs recrudescence. Very challenging. Other species also not mentioned. The paper would benefit from at least a mention of the species relevant to their recommendations.*

We agree about the important differences for *P. vivax* and have made the following addition to the discussion: “Our work focuses specifically on efficacy trials for participants with *P. falciparum*, as this is the species with the most documented resistance and is responsible for the majority of global burden. Evaluating antimalarial efficacy against other species is additionally complicated by differences in parasite lifecycle. For example, recurrence in patients with *P. vivax* can be due to either recrudescence, new infection, or relapse. The TPPs outlined here are a good starting point for development of *P. vivax*-specific guidance, but additional considerations are needed to design and validate genotyping and statistical approaches to molecular correction for *P. vivax* trials.”

*5) Line 342, mentions “weakness of older methods” – but authors do not describe nor discuss what these are.*

Some discussion of the weaknesses of older methods was previously included in the introduction, but we agree with the reviewer that explicitly laying out the issues negatively affecting the accuracy of currently recommended methods would provide improved context for the reader and better motivate the TPP. Therefore, we have added an overview of these weaknesses in the new summative Table 1, and additionally include a new supplemental table (below) to provide more detailed context, now cited in the introduction section.

| **Supplemental Table 1. Issues negatively affecting accuracy of TES genotyping classification using currently recommended methods.** All issues listed here have a greater effect on efficacy outcomes as the proportion of study participants with recurrent infection increases (i.e. when transmission and/or recurrence rates are high). | | | |
| --- | --- | --- | --- |
| **Issue adversely affecting estimates of treatment failure** | **Effect on treatment failure estimate** | **Factors increasing magnitude of effect** | **Proposed solutions** |
| Alleles from reinfection on Day of recurrence match those on Day 0 by chance | Overestimation | - Fewer loci  - Lower diversity of loci  - More related parasite population  - Higher COI | - Increase number and diversity of loci  - Analysis methods accounting for allele frequency, relatedness of parasites, and COI of infections |
| False positive alleles | Overestimation | - Poor specificity, e.g. due to PCR artifacts generating when amplifying repetitive regions  - Higher probability of false positive alleles matching true alleles | - Increase specificity of lab methods  - Use lab methods where false positive alleles are less likely to be the same as true alleles  - Analysis methods accounting for false positive alleles |
| False negative alleles | Underestimation | - Higher COI (resulting in more low abundance alleles)  - Larger range of allele size (PCR amplification bias for shorter alleles)  - Limited dynamic range for detection  - Misidentification of alleles due to wrong sizing of length polymorphisms | - Amplification of targets where alleles have similar length  - Increase sensitivity for detecting low abundance (minority) alleles  - Analysis methods accounting for false negative alleles  - Improve sizing or switch to NGS methods |
| Subjective genotyping methods | Inconsistent / irreproducible results | - High rates of unpredictable artifact generated by PCR of repetitive regions  - Difficulty in determining true alleles from artifact when using electrophoresis, requiring “expert” interpretation | - Lab methods with fewer and more tractable false positive alleles  - Automated analysis pipelines for data analysis |
| Inconsistent approach to analysis | Inconsistent / irreproducible results | - Simpler, match counting algorithms result in biased, inconsistent results  - Some methods difficult to implement | - Easy to use, accurate, and validated analysis tool to calculate treatment failures  - Proper accounting for uncertainty  - Automated collation of reporting data |

*6) Advice provided that sequencing, i.e., amplicon-based, would be more accurate and cost effective compared to older methods and capillary designed experiments etc however no specific information was provided on this and evaluations of NGS vs capillary electrophoresis.*

We thank the reviewer for this comment. Recent studies have compared amplicon-based NGS assays and capillary electrophoresis (CE), and the data showed that NGS assays were highly sensitive in detecting minority clones, and they provided more consistent results between different markers compared to CE (Cantoreggi et al, 2025; Schnoz et al, 2024). Moreover, due to their multiplexing capacity, the NGS assays have in practice become more cost-efficient compared to CE (Holzschuh et al, 2025, Aranda-Diaz et al, 2025). These references have been added to relevant section of the discussion.

*7) Line 384, “To date, four statistical methods have been proposed, three of which have accompanying software packages.(14,15,20,21)”. . This paragraph assumeds knowledge of these methods and packages so it would be useful to include named and brief details of the packages.*

Thank you for noting the omission of a description of these methods. We have added the following text: “Briefly, three of these methods are statistical inference approaches where likelihoods are derived from functions of the frequencies of observed alleles, and one method is a non-parametric, statistical threshold-based approach where the empiric null distribution of genetic distances is derived from pairs of unrelated Day 0 samples.”

*8) Limited details of the simulation methods or underlying data provided*

We have expanded the relevant section in the methods (new version below) and included the code for simulations as supplementary material.

“We performed simple simulations to provide practical guidelines on the number and diversity of genetic loci required to distinguish recrudescence from new infection at a level of accuracy listed in the TPP. Details of the simulations including code and results are provided. In brief, we used a grid of approximately 4000 (2^12) heterozygosity (He) values with corresponding randomly generated population allele frequencies; these frequencies were repeated for each locus. For each constructed panel and each complexity of infection (COI) value, we simulated 10,000 Day 0 samples and 10,000 recurrent samples representing new infections. A second set of recurrent samples representing recrudescences was constructed by replacing one of the newly infecting strains with a strain from the corresponding Day 0 infection. Genotyping errors were added to each sample, assuming 90% probability of allele detection and a false positive rate of 2%. We then used the *asterTES* package to classify each Day 0 – Day of recurrence pair as a recrudescence or a new infection.(20) Classification results of new infection pairs were used to assess specificity and classification results of recrudescent pairs to assess sensitivity. The process was repeated until the desired performance characteristics were achieved (95% specificity and 95% sensitivity). More details and simulation code are included in the supplemental materials. Classification results were then used to calculate specificity and sensitivity; the smallest He value to achieve the desired performance level is presented.”

Reviewer #2: Manuscript:

 *Target product profiles of laboratory and data analytical frameworks for genotyping to monitor antimalarial efficacy*

 This manuscript addresses an important topic related to improving the accuracy and reliability of genotyping approaches used in Therapeutic Efficacy Studies (TESs) for antimalarial drugs, specifically for Plasmodium falciparum. Strengthening the framework used to distinguish recrudescence from reinfection is critical for accurate treatment efficacy and policy decisions. The manuscript provides guidelines for laboratory and analytical methods aimed at standardising genotyping practices.

 *It appears that these guidelines were generated from meetings across collaborators in US, who constitute most of the authors. While the work reflects considerable expertise in assay development and statistical analysis, it is surprising that representation from endemic regions is limited. Only a few contributors from Africa are included, and none from Asia, where monoclonal infections present additional challenges for TES. Broader input from endemic settings and independent research groups would strengthen the manuscript’s relevance*.

We are in full agreement with the reviewer's perspective and acknowledge disparities in the geographical representation of the experts' affiliations. We contacted a broad and diverse group of experts at the beginning of the project, attempting to capture as many backgrounds and geographical representativeness as possible. Not everyone on the list agreed to participate due to the time commitment and ability to engage fully in this process, and we ended up with the current participant list as reflected in the paper. We do acknowledge overrepresentation of researchers in the Global North as a major limitation of the field, a reflection of historical imbalances. However, we are optimistic that the laboratory advances that we review and discuss in this perspective will tilt the balance towards performing most molecular corrections in endemic countries. Many of the authors are, and have been, involved in years-long and successful efforts to build and support molecular surveillance capacity in Africa. Moreover, throughout the manuscript we stress the importance of transparent, reproducible, and open-source statistical methods that can lower the barriers to performing the full genotyping and statistical analysis pipelines in endemic countries (see for example the "access and usability" row in Table 5).

*Areas where the manuscript could be improved:
 Geographic representation and epidemiological considerations: The manuscript briefly mentions other geographic and epidemiological challenges, but a more thorough discussion of the implications for monoclonal versus polyclonal infections in different regions, and situations of outbreaks or (re) introductions would strengthen the work.*

The reviewer is correct that the manuscript intentionally focuses on high transmission regions, since nearly all *P. falciparum* efficacy monitoring occurs in these areas, new infections are much more likely to occur (resulting in a higher proportion of cases requiring genotyping), and higher COI greatly exacerbates issues with accurate classification (see details in Supplemental Table 1, added in response to Reviewer 1’s comments above). In very low transmission areas with clonal propagation and limited population diversity, the reviewer is correct that it can be difficult to distinguish recrudescence from new infection. Though already addressed by the TPP in discussions of ensuring adequate genetic diversity, we have added this explicitly in the discussion section,

“In areas of very low transmission, a lack of population genetic diversity can also present difficulties for genotyping classification. “

*Practical pipeline guidance: The manuscript would benefit from a more pipeline-oriented approach that guides users in practical decisions based on laboratory capacity (e.g., MinION vs. Illumina, capillary), regional transmission intensity, and other situational factors. Including flowcharts or decision tables would make the guidance more actionable and improve readability.*

As with reviewer 1 (comment #3) we completely agree that detailed protocols outlining practical decisions such as those listed are a near term goal. However, these need to be determined by appropriate stakeholders (such as the WHO) in the context of standardized benchmarking and validation of laboratory and analysis methods. These data are not yet available, but we hope that outlining proposed criteria in this TPP is a helpful first step.

*Tables: Given the number of tables, the manuscript can become difficult to navigate. Some tables are lengthy and include information that may be obvious to the target audience. For example, Table 1 could be condensed into a few sentences. Tables 3 and 5 contain information that is not critical and could be trimmed. Simplifying tables and combining them with workflow illustrations could improve usability and readability.*

We appreciate the comment and agree that the original version of this manuscript was not as accessible as we hoped. Based on this feedback and feedback from the other reviewer, we have added a new summary, key-messages table (Table 1). We believe that this addition helps ground the manuscript and orient the reader early on. Key points from the other tables, in some ways the “main product” of this manuscript, are outlined in the text. We are also moving one of the more technical tables (listing numbers of loci and heterozygosity) to the supplement to keep the main tables focused on key points and the TPP itself.

*Economic considerations: Including example costs for personnel, equipment, and consumables across platforms in representative endemic countries would provide a practical context for implementation.*

We agree with reviewer that economic considerations are important. These are included as considerations in the TPP, but as with specifics for laboratory methods, costs need to be appropriately validated in a standardized manner to be reliable. Several authors have gone through this exercise, and it is not as straightforward as it might appear. Fortunately, the WHO is currently working on formal costing of several methods via a standardized tool in concert with FIND, and these results when available should indeed be very helpful in guiding next steps.

 *This work has strong potential to contribute to the field by guiding the development of robust, standardised TES genotyping pipelines. With additional attention to geographic representation, methodological limitations and cost, and practical implementation, the manuscript could provide a solid foundation for establishing community standards.*

We appreciate the reviewer’s thoughtful comments and constructive feedback.
